# Evaluating the effectiveness of the eHealth-faciLitated SMILe–integrated care model on treatment burden, quality of life and graft-versus-host disease after allogeneic stem cell transplantation: results of two European randomized controlled implementation studies

**DOI:** 10.1007/s00277-026-07333-1

**Published:** 2026-10-24

**Authors:** Lynn Leppla, Sabine Valenta, Kris Denhaerynck, Anja Schmid, Janette Ribaut, Alexandra Teynor, Thomas Meyer, Claudia Wehr, Robert Zeiser, Sabine Gerull, Jörg Halter, Jakob Passweg, Sabina De Geest

**Affiliations:** 1 Department of Medicine I, Medical Center, Faculty of Medicine, University of Freiburg, Freiburg, Germany; 2 Department of Nursing, Medical Center, University of Freiburg, Freiburg, Germany; 3 Institute of Nursing Science, Department Public Health, University of Basel, Basel, Switzerland; 4 Nursing Science, Institute of Health Sciences, Medical Faculty, University of Tübingen, Tübingen, Germany; 5 Faculty of Computer Science, Augsburg University of Applied Sciences, Augsburg, Germany; 6 Department of Hematology, Cantonal Hospital Aarau, Aarau, Switzerland; 7 Department of Hematology, University Hospital Basel, Basel, Switzerland; 8 Academic Center for Nursing and Midwifery, Department of Public Health and Primary Care, Katholieke Universiteit (KU) Leuven, Leuven, Belgium

**Keywords:** eHealth, Integrated care model, Allogeneic stem cell transplantation, Hybrid effectiveness-implementation study, Graft-versus-host disease, Quality of life

## Abstract

The SMILe Integrated Care Model (SMILe–ICM) has previously demonstrated feasibility, successful implementation, and promising effects on survival, re-hospitalization, and cost-effectiveness following allogeneic stem cell transplantation (alloSCT). To complement these findings and provide a more comprehensive evaluation of the intervention’s effects, we evaluated patient-reported and clinical secondary outcomes from two RCTs. Data from two hybrid effectiveness-implementation RCTs conducted at two European alloSCT centers (Germany, Switzerland) were analyzed. Adult alloSCT recipients were randomized 1:1 to usual care (UCG) or the SMILe–ICM, a multicomponent intervention combining eHealth with advanced practice nurse-led self-management support. Treatment burden was assessed using the Patients’ Experience of Treatment and Self-Management (PETS 2.0), HRQoL using the EQ-5D-5 L, and GvHD outcomes using EBMT-registry data. Analyses followed an intention-to-treat approach. The current study examined data from 152 patients (76 SMILe–ICM, 76 UCG). Treatment burden was lower in the SMILe–ICM group, with significant differences in five dimensions. Both groups’ HRQoL improved over time without significant between-group differences. The cumulative incidence of acute GvHD was lower in the SMILe–ICM group than in the UCG (34.2% vs. 55.3%; HR 0.54, 95% CI 0.33–0.88; *p* = 0.01); chronic GvHD did not differ significantly (*p* = 0.64). SMILe–ICM reduced treatment burden and was associated with a lower cumulative incidence of grades II–IV acute GvHD, while HRQoL and chronic GvHD did not differ significantly between groups. The acute GvHD finding warrants further investigation to determine whether and through which pathways integrated APN-led survivorship care may influence post-alloSCT clinical outcomes.

**Supplementary Information:**

The online version contains supplementary material available at https://doi.org/10.1007/s00277-026-07333-1.

## Introduction

Although major advances in donor selection, conditioning regimens, and supportive care have substantially improved survival after allogeneic stem cell transplantation (alloSCT) [1–3], survivors remain at increased risk of graft-versus-host disease (GvHD), infections, treatment-related toxicities, and multimorbidity [4, 5]. Compared with the general population, even years after transplantation, these complications impair health-related quality of life (HRQoL), exacerbate treatment burden, and increase mortality risk [4–7].

As survivorship care becomes increasingly complex, patients and caregivers face a widening range of behavioral tasks, such as symptom monitoring, infection prevention, medication adherence, physical activity, and dietary recommendations [7–9]. And as care needs grow, post-alloSCT follow-up evolves from managing acute transplant complications towards proactive integrated care for patients with chronic healthcare needs [10]. However, current care models remain largely acute care-driven, commonly providing insufficient attention to care continuity, care coordination, and sustained self-management [10, 11]. As a result, despite strong links with GvHD, re-hospitalization, morbidity, and mortality, non-adherence to immunosuppressive medication and infection prevention measures remains common [8, 12–15]. Therefore, an integrated care model (ICM)—one that strengthens continuity of care while combining coordinated multidisciplinary follow-up with proactive self-management support—represents a promising strategy to improve long-term alloSCT outcomes [10].

**Fig. 1:**
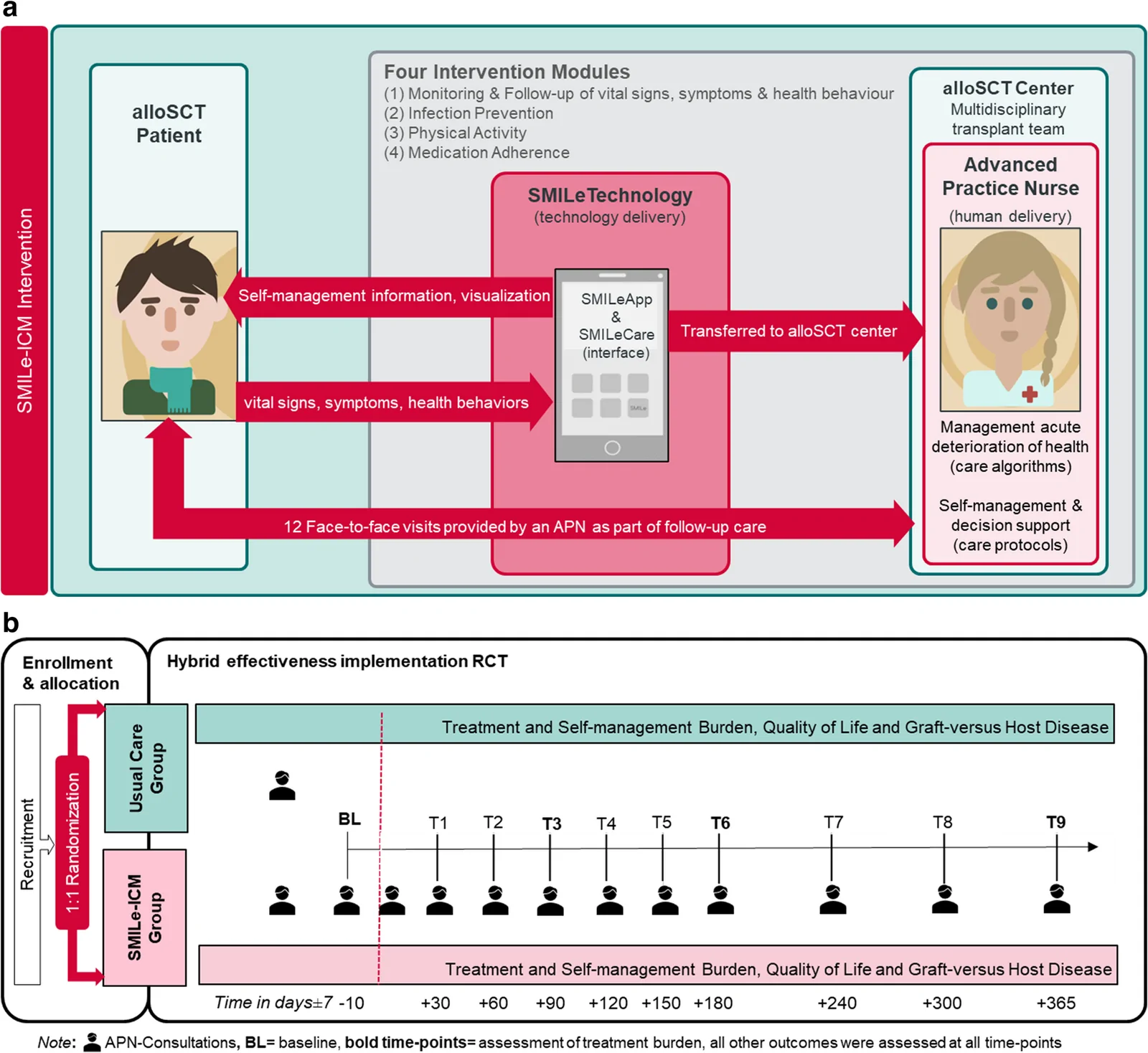
**a**: The SMILe-integrated care model [15]. **b**: APN-consultations and measurement time-points, adapted from De Geest et al. [18]. Note: alloSCT= allogeneic stem cell transplantation center; APN= Advanced Practice Nurse

To address these challenges, we developed the allogeneic SteM cell transplantatIon faciLitated by eHealth Integrated Care Model (SMILe–ICM), an Advanced Practice Nurse (APN)-led integrated chronic care model combining evidence-based self-management support, care coordination, continuity of care, and eHealth-facilitated remote monitoring [16]. Based on in-depth contextual analyses, the SMILe–ICM was initially developed at University Hospital Freiburg (UKF; Germany) [11, 16–18], then further developed and adapted to University Hospital Basel (USB, Switzerland) [18–21]. The SMILe–ICM combines structured APN follow-up with digital symptom and medical parameter monitoring, alongside APN-delivered self-management interventions to support infection prevention, medication adherence, and physical activity. Its combination of human and technological components facilitate self-management support, early complication detection, and individualized care coordination throughout the first post-transplant year (see Fig. 1a/b).

We evaluated SMILe–ICM outcomes from linked but separate RCTs at the UKF [22] and USB [19, 23] alloSCT centers. Although both trails were underpowered regarding their primary outcomes (*costs*, *cost-effectiveness*, *re-hospitalizations*), they provided promising effectiveness signals. At UKF, the SMILe-ICM was associated with improved *survival* (88% vs. 80% in the usual care group (UCG) and favorable *cost effectiveness* at UKF (€6,742/life-year gained, dominant cost-effectiveness for patients living alone) [22], whereas at USB, the intervention prolonged the time *to first rehospitalization* (median 86.5 days vs. 56.0 days UCG) [23].

Building on this initial evidence, to determine pathways via which integrated APN-led survivorship care models to improve long-term patient outcomes, we focused on both centers’ SMILe–ICM secondary outcomes. Specifically, we investigated *treatment burden*, *HRQoL*, and three GvHD parameters: *cumulative incidence*, *maximum severity* and *time to onset*. We hypothesized that SMILe–ICM patients would experience lower *treatment burden*, better *HRQoL*, and comparable *GvHD incidence*, but lower *maximum GvHD severity* and a longer *time to GvHD onset* than their UCG counterparts [19].

## Methods

### Patient and public involvement

Following implementation science methodology, a patient representative was included in the project’s overall steering group to support the design and implementation phases. During the dissemination phase, the participating centers’ patient advisory boards were also actively involved.

### Design

We used data from the UKF’s and USB’s linked, yet separate, non-blinded hybrid effectiveness-implementation RCTs [22, 23]. For both studies, we allocated patients 1:1 to the usual care group (UCG) or the SMILe–ICM intervention group (IG). Both study protocols were registered (DRKS00020347 and NCT04789863), and both research groups adhered strictly to the Declaration of Helsinki ethical principles. Each received ethical approval from its respective ethics committee (UKF: EK 309/19; USB: EKNZ 2021 − 00202). This report follows the CONSORT 2025 guidelines [24].

### Settings, samples and eligibility criteria

Both study hospitals are among the largest academic hospitals in their regions, typically treating 100–120 adult alloSCT cases annually. Relevant contextual information regarding perceived care needs, practice patterns, stakeholders’ openness to technology, our basis for developing/adapting the SMILe–ICM and our chosen implementation strategies are reported elsewhere [11, 21]. Both centers’ recruitment and data collection lasted roughly 29 months (UKF: 2/2020–7/2022; USB: 4/2021–9/2023).

The two studies’ sampling procedures were similar. Every adult patient (≥ 18 years) scheduled for alloSCT was eligible if: any part of their first-year post-alloSCT follow-up care was scheduled at UKF or USB; they had adequate German language proficiency; they were capable of planning and performing self-management activities independently (defined as ECOG ≤ 4 in the German center); they had home internet access or a sufficient mobile data contract; and they had the mental, physical and psychological capacities to provide written informed consent regarding study participation and related data protection. Any who required a second alloSCT within the first year were excluded.

### Usual care vs. intervention

While the SMILe–ICM intervention was very similar for both study sites (see exceptions below), the usual care structures between the two study sites differed notably.

Usual care patients received one multiprofessional (i.e., APN, nurses, physician) structured pre-transplant counseling session to discuss the procedure, risks, and post-transplant care, normally 1–3 months prior to alloSCT. At both centers, patients were hospitalized approximately ten days pre-transplant and remained hospitalized for 25–30 days afterwards. However, whereas UKF patients could choose to be discharged to a rehabilitation clinic for roughly three weeks post-transplantation, USB discharged all directly to their homes. For the first year post-alloSCT, both centers had comparable follow-up structures: One to three follow-up visits were planned per week for three months, tapering to weekly/bi-weekly visits by six months, then to annual visits by year five. When possible or necessary (e.g., for patients living farther away) follow-up was divided between a local hematologist and the transplant center. Both centers routinely started tapering immunosuppressive medication around day + 100 post-alloSCT.

#### SMILe–ICM patients

Participants allocated to the intervention group (IG) received usual care plus the SMILe–ICM. The intervention combined (1) eHealth-supported remote monitoring with (2) structured APN-led survivorship care delivered by master’s-prepared alloSCT-specialized APNs (Fig. 1).

1. The eHealth component consisted of two platforms: the patient-facing SMILeApp and the clinician-facing SMILeCare. Patients used the SMILeApp to document symptoms, selected medical parameters and medication adherence daily. If patients did not enter data daily, the APN team proactively contacted them after four days. For patients experiencing a high documentation burden, a reduced minimum frequency of once-weekly reporting was agreed upon individually. Patient-reported data were securely transmitted to the SMILeCare platform, enabling the APN team to continuously review patients’ health status between scheduled consultations. Predefined thresholds, established during the systematic development of the SMILe-ICM [16], guided APN follow-up according to a monitoring and response protocol, including watchful waiting, patient contact, clinical assessment, and, when indicated, consultation with the transplant physician (Supplementary Table 1). The APNs also coordinated further assessment and management within the multidisciplinary transplant team, involving the responsible transplant physicians whenever medical decision-making or treatment adjustments were required.
2.  Moreover, APNs conducted twelve structured consultations per patient at predefined timepoints, beginning with the pre-transplant counseling session (1–3 months prior to alloSCT at UKF, d-10 at USB) and continuing throughout the first post-transplant year (Fig. 1b). Post-transplant consultations were aligned with routine outpatient follow-up appointments–initially weekly, then monthly for clinically stable patients. During these consultations, the APN team delivered evidence-based intervention modules addressing symptom and medical parameter monitoring, infection prevention, medication adherence, and physical activity. Based on remotely monitored data and individual needs, APNs provided tailored education, behavioral counselling, self-management support and care coordination. As integral members of the multidisciplinary transplant team, the APNs facilitated communication between healthcare professionals, promoted continuity of care, and coordinated timely interventions when clinically indicated.

### Variables and measurement

This paper reports the combined secondary outcomes of both studies [19, 22], i.e., *treatment burden*, *HRQoL*, and *GvHD incidence*, *maximum grade*, and *time to onset*. Demographic characteristics (*age*, *sex*, *education level*, and *living status*) and conditioning regimen were assessed at baseline (BL) using medical records and patient-reported data. *Conditioning intensity* was classified using the Transplant Conditioning Intensity (TCI) score [25]. For details of all study variables, measurement instruments, and assessment time points, see Table 1.

**Table 1:** Study variables, measurement instruments and assessment time points

Variable/ Outcome	Measurement/ Instrument	Description	Time points
Demographic characteristics	Medical records and patient report	Age, sex, education level, living status	Baseline (T0)
Conditioning regimen	Transplant Conditioning Intensity (TCI) score [1]	Classification of conditioning intensity; accounts for individualized dosing	Baseline (T0)
APN-delivered intervention fidelity	Attendance; intervention protocols	Attendance at scheduled APN consultations; proportion of planned Behavior Change Techniques (BCTs) delivered	T1-T9
eHealth engagemen	SMILeApp usage data	Post-transplant observation period (days), total number of SMILeApp entries, and number of weeks without a SMILeApp entry	From alloSCT - day + 365 post.transplant
Treatment and self-management burden	PETS 2.0 questionnaire [2]	60-item instrument covering 14 domains, 12 multi-item burden scales and 2 single-item indicators, measuring the burden of one aspect of chronic illness treatment on a 4- or 5-point Likert-type response scale for a 4-week recall period. Raw domain scores are transformed into a standardized 0–100 metric. Three dimensions—exercise/physical therapy, diet and difficulty with healthcare services—were reverse scored from positive to negative, i.e., higher scores indicate greater treatment burden.	T0, T3, T6, T9
Health-related quality of life (HRQOL)	EQ-5D-5 L index [3]	Five-dimensional health state classification: (1) mobility (“Do you have problems walking around?”), (2) self-care (“Do you have problems washing or dressing yourself?”), (3) usual activities (“Do you have problems performing your usual activities?”), (4) pain/discomfort (“Do you have pain or discomfort?”), and (5) anxiety/depression (“Do you feel anxious or depressed?”). Each dimension is rated on a five-point Likert scale (level 1 = no problems; level 5 = extreme problems). Levels are coded as single-digit numbers and combined into a 5-digit profile representing the individual’s overall health state (e.g., 11111). This health-state profile is then converted into a single summary measure, the EQ-5D index value. The index value reflects the relative desirability of the health state according to country-specific population preferences and ranges from < 0 “worse than death” to 1 “very good health”.	Baseline (T0) and T1-T9
Health-related quality of life (HRQOL)	EQ Visual Analogue Scale (EQ-VAS)	Individuals rate their overall perceived health state ranging from 0 = worst to 100 = best perceived HRQOL	Baseline (T0) and T1-T9
Acute GvHD	EBMT registry data	Organ involvement staged and used to determine an overall grade of 0–IV [4]; grades 0–I = absent and grades II–IV = present acute GvHD	T1-T9
Chronic GvHD	EBMT registry data	Organ involvement scored 0–3 and used to determine overall severity as mild, moderate, or severe according to the 2014 NIH consensus criteria [5]; categorized as absent or present	T1-T9

Note. *BCT* Behavior Change Techniques, *EBMT* European Society for Blood and Marrow Transplantation, *EQ-5D-5 L* EuroQol five-dimensional five-level questionnaire, *EQ-VAS* EuroQol visual analogue scale, *GvHD* graft-versus-host disease, *HRQOL* health-related quality of life, *NIH* National Institutes of Health, *PETS* Patient Experience with Treatment and Self-management, *TCI* Transplant Conditioning Intensity

Intervention fidelity within the SMILe-ICM group was assessed across the eHealth and APN-delivered components. Engagement with the eHealth component was assessed using previously reported SMILeApp data [26]. Participants were included in this analysis if they had ≥ 1 pre-alloSCT entry, ≥ 10 post-alloSCT entries through day + 90, and thereafter a total number of entries equivalent to at least weekly reporting until the end of the intervention. eHealth engagement was characterized by the post-alloSCT observation period (days), total number of SMILeApp entries, and number of weeks without an App entry. Fidelity to the APN-delivered component was assessed by (1) participant attendance at scheduled APN consultations and (2) intervention delivery based on the predefined Behavior Change Techniques (BCTs). Intervention documentation was reviewed to determine the proportion of planned BCTs that were delivered.

Treatment and self-management burden was measured using the Patients’ Experience with Treatment and Self-management questionnaire (PETS 2.0) [27]. As no German version was available, the questionnaire was translated and linguistically validated according to WHO recommendations [28]. PETS 2.0 covers fourteen domains assessing specific aspects of treatment burden. Domain scores were standardized to a range of 0–100, with higher scores indicating greater treatment burden.

Health-related quality of life (HRQoL) was assessed using the EQ-5D-5 L and the EQ Visual Analogue Scale (EQ-VAS) [29]. The EQ-5D-5 L index value was calculated using the German value set [30], which ranges from − 0.18 to 0.95. The EQ-VAS ranges from 0 (worst imaginable health state) to 100 (best imaginable health state) [29].

Graft-versus-host disease (GvHD) incidence, maximum observed grade/severity, and time of onset were extracted from both centers’ EBMT registry datasets. For acute GvHD, organ involvement was staged and translated into an overall grade of 0–IV [24]. For chronic GvHD, individual organ involvement was scored from 0 to 3 and used to determine overall severity as mild, moderate, or severe according to the 2014 NIH consensus criteria [31]. As mild acute GvHD is usually tolerated, we classified overall grades of 0–l as “no acute GvHD” and grades Ⅰ-Ⅳ as “GvHD present.” Chronic GvHD was classified as absent (0) or present (mild, moderate, severe) [31].

### Sample size and randomization

This study represents a secondary analysis of two RCTs evaluating the SMILe–ICM. As previously reported, sample size calculations for both trials were based on their respective primary outcomes [22, 32]. At both study sites, the project leaders (LL, SV) consecutively screened all patients scheduled for alloSCT according to predefined inclusion and exclusion criteria. Eligible patients who provided written informed consent were randomly allocated (1:1) to either the UCG or the SMILe–ICM group. Randomization was stratified according to previously identified factors known to influence study outcomes, including *age* (< 65 or ≥ 65 years), *sex* (female or male), and *living status* (alone/not alone). An automated randomization procedure was performed using secuTrial^®^, a web-based, GCP-compliant clinical data management system. Although randomization and group allocation were conducted independently from the clinical team, further blinding was not feasible.

### Statistical analyses

The two studies’ secondary outcome data were combined to perform an intention-to-treat analysis using SAS 9.4 statistical software (SAS Institute Inc, Cary, NC). Descriptive statistics were applied as appropriate for all variables.

#### Treatment and self-management burden and Health-Related Quality of Life (HRQoL)

Regarding *Treatment Burden* and *HRQOL*, UCG-vs.-SMILe–ICM-group differences were analyzed over time using mixed regression analyses, allowing a random intercept for each patient. Missing data were not imputed; however, inclusion of the random effect implies a missingness at random adjustment, conditional on between-patient differences.

#### Graft-Versus-Host Disease (GvHD)

The occurrence of acute and chronic GvHD over time was estimated using the cumulative incidence function, while accounting for the presence of competing risks (i.e., death prior to GvHD occurrence, prior donor lymphocyte infusion). Hazards ratios were calculated using Fine and Gray proportional hazards regression and adjusted for center. Patients who dropped out were censored.

## Results

### Demographic and clinical characteristics

**Fig. 2:**
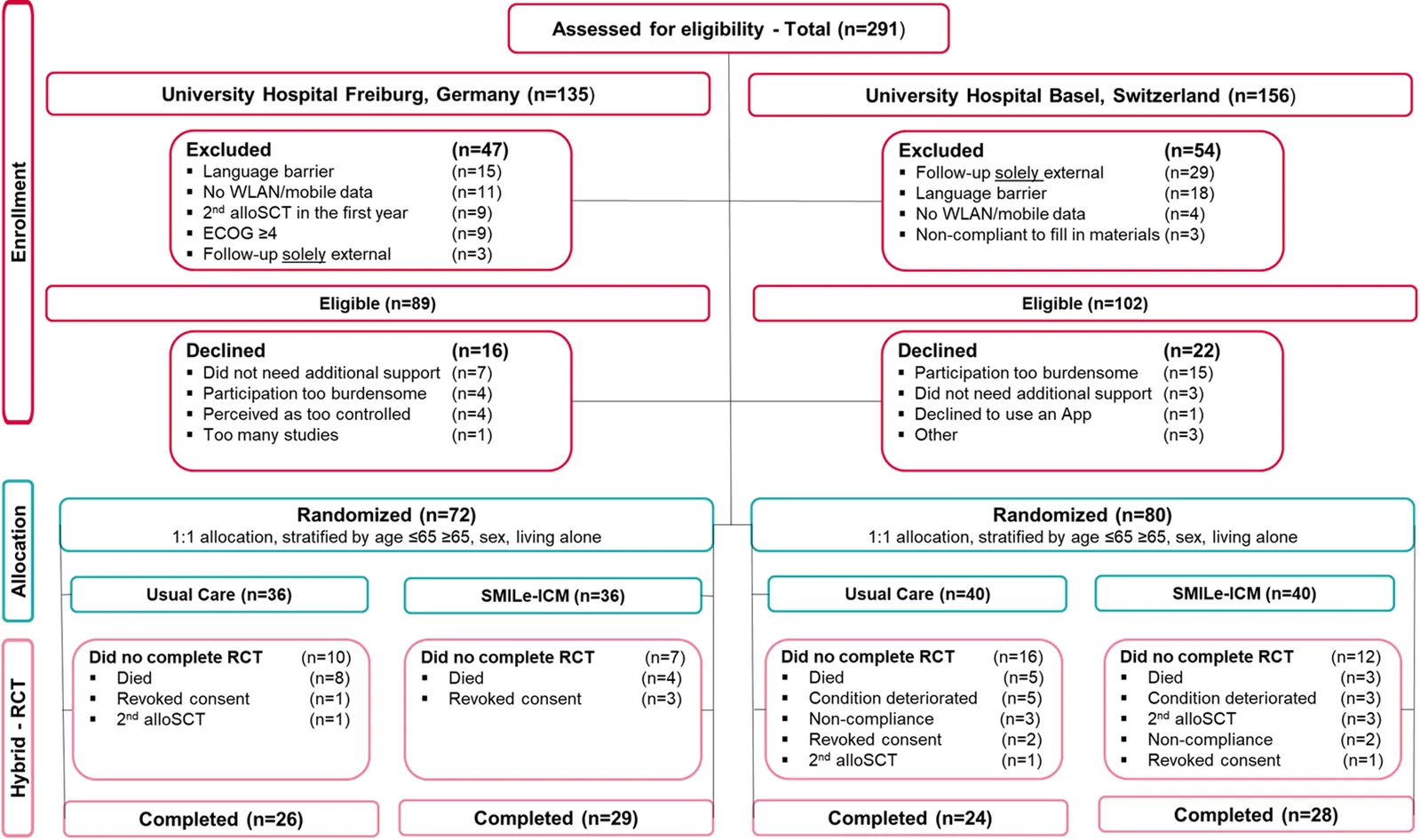
Flow-chart of the two hybrid-RCTs at both centers

Of the two centers’ 152 patients (UKF:72; USB:80), 76 each were assigned to the UCG and SMILe–ICM group (see Fig. 2). As Table 2 shows, the groups’ BL characteristics were comparable.

**Table 2:** Patient demographics

Variable	Total	UCG	SMILe-ICM
Total (N)	152	76	76
Sex (N, %)			
Male	96 (62.75)	48 (63.16)	48 (62.34)
Female	56 (37.25)	28 (36.8)	28 (37.6)
Age (years)			
Median	58	58	59
Min/Max (IQR)	19–76 (15)	21–73 (17)	19–76 (14)
Diagnosis (N, %)^1^			
Myelodysplastic/myeloproliferative neoplasms	40 (26.14)	11 (14.47)	29 (37.66)
Acute leukemias	74 (49.02)	41 (53.95)	33 (44.16)
Lymphomas	22 (14.38)	12 (15.79)	10 (12.99)
Others	16 (10.46)	12 (15.79)	4 (5.19)
Conditioning regimen according to TCI-Score ^2^ (N, %)			
Low	59 (39)	32 (42)	27 (36)
Intermediate	80 (53)	36 (47)	45 (58.5)
high	13 (8)	8 (11)	4 (5.5)
Status of relationship			
Living alone (N, %)	26 (16.99)	11 (14.47)	15 (19.48)
Educational level (N, %)			
Basic and lower secondary education	87 (57.52)	41 (53.95)	46 (61.04)
Higher secondary education	5 (3.27)	3 (3.95)	2 (2.6)
Tertiary education	57 (37.25)	30 (39.47)	27 (35.06)
Missing	3 (1.96)	2 (2.63)	1 (1.3)

Note: ^1^ We classified the diagnoses following the WHO recommendations [6, 7]. They are classified by: (1) myelodysplastic and myeloproliferative neoplasms: Chronic myeloid leukemia, myeloproliferative syndromes, myelofibrosis, primary myelofibrosis, myelodysplastic syndrome; (2) acute leukemias: acute lymphocytic leukemias, acute myeloid leukemias; (3) lymphomas: B-cell-lymphomas, non-Hodgkin lymphomas, multiple myeloma; (4) others: severe aplastic anemia, sickle cell anemia;^2^ *TCI score* Transplant Conditioning Intensity Score

### Fidelity to the eHealth and APN-delivered intervention components

Among SMILe–ICM participants included in the previously reported eHealth engagement analysis (*n* = 67) [26], the median post-transplant observation period was 359.0 days (IQR, 339.5–365.0), with a median of 251.0 SMILeApp entries (IQR, 160.5–324.5). During this period, the median number of weeks without a SMILeApp entry was 3.0 (IQR, 1.0–6.0). Intervention fidelity with the APN-delivered interventions was high at both centers. At UKF, 460 APN visits were delivered (mean duration, 43 min), with five visits not delivered as planned due to patients’ poor health status (1.1%). At USB, 489 visits were conducted, with seven not delivered for the same reason (1.4%). Fidelity to the delivered BCTs ranged from 60% to 95% across centers, with the highest fidelity observed during the first and final visits. Deviations from the protocol mainly reflected repetition of BCTs or the unavailability of caregivers.

### Treatment burden

**Fig. 3:**
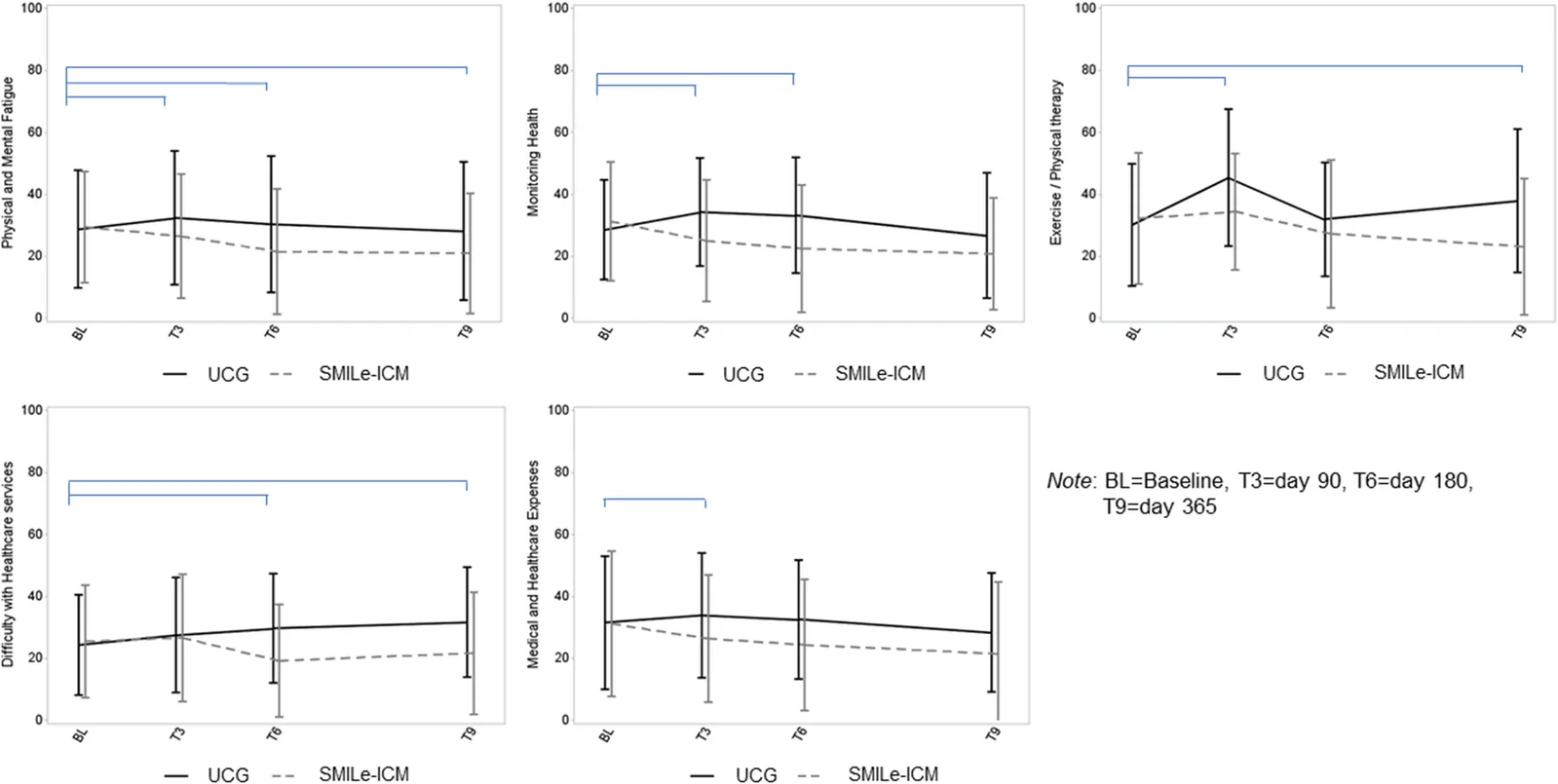
Treatment burden domains with significant between-group and time effects. *Note.* Blue brackets indicate statistically significant differences between time points and/or between groups (*p* < 0.05)

Baseline treatment burden was comparable between groups. During follow-up, SMILe–ICM patients consistently reported lower treatment burden across all domains than the UCG (Fig. 3). Significant intervention effects favored the SMILe–ICM in five domains: *physical and mental fatigue* between BL and T3 (*p* = 0.03), and at T6 (*p* = 0.0008), and at T9 (*p* = 0.02), *Monitoring health* between BL and T3 (*p* = 0.019), and at T6 (*p* = 0.005), *Exercise or physical therapy* between BL and T3 (*p* = 0.002) and T9 (*p* = 0.004), *Difficulty with healthcare services* between BL and T6 (*p* = 0.003) and at T9 (*p* = 0.023), and *Medical and healthcare expenses* between BL and T6 (*p* = 0.031). No significant differences were observed for the remaining domains. Beneficial intervention effects were sustained until d + 365 regarding three domains (Fig. 3 shows all domains with significant differences between groups and/or time points).

### HRQoL

**Fig. 4:**
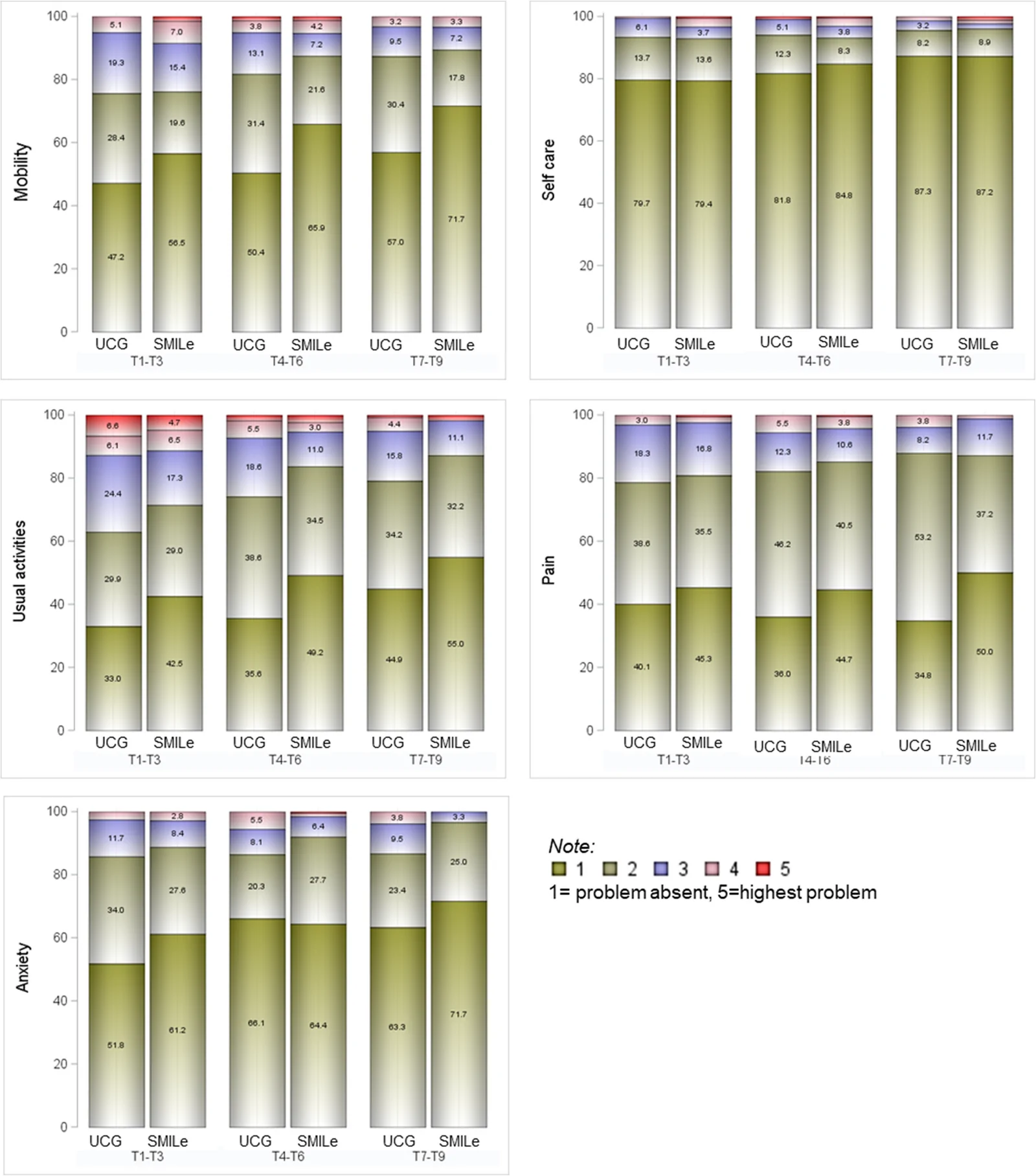
EQ–5D–5 L dimensions per time point and group

HRQoL dimensions are depicted separately in Fig. 4. Both groups’ BL index values were above 0.84 (Supplementary Table 2). After T1 (d + 30) yielded both groups’ worst mean index values–0.81 (UCG) and 0.82 (IG)–the UCG regained its BL level at T5 (d + 150) and IG at T4 (d + 120). The UCG reached T9 (d + 365) at 0.87 (UCG), the IG at 0.88. We found no significant intergroup divergences. VAS scores were comparable at BL (UCG 56%, SMILe–ICM 63%). The groups showed parallel improvements, reaching 77% (UCG) and 80% (SMILe-ICM) at d + 365 (Supplementary Fig. 1).

### Graft versus host disease

**Fig. 5:**
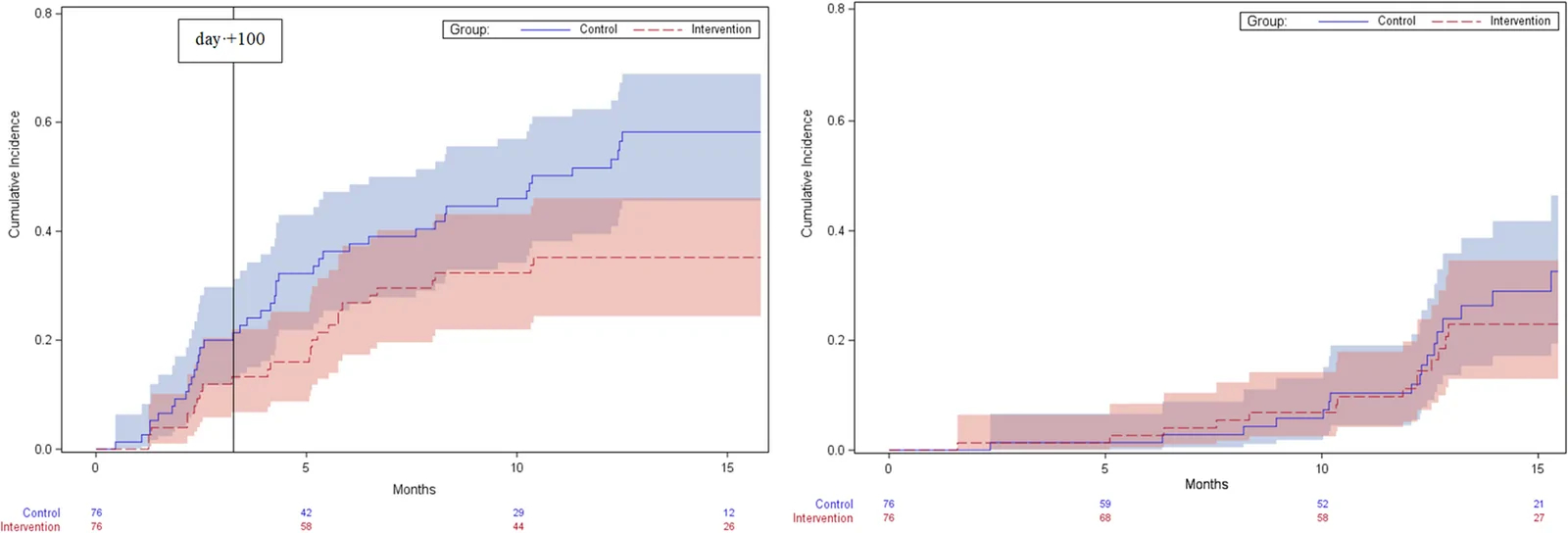
Cumulative incidences of acute and chronic GvHD. Note. Cumulative incidence of acute and chronic GvHD over time in the UCG (solid blue line) and SMILe-ICM/IG (dashed red line). Shaded areas indicate 95% confidence intervals. Numbers of patients at risk are shown below the x-axis at selected time points

Cumulative incidence of the first-year acute GvHD reached 55.3% in the UCG and 34.2% in the SMILe–ICM group (hazard ratio 0.54, 95% CI 0.33–0.88; *p* = 0.01). At day + 100, the cumulative incidence of acute GvHD was 22% in the UCG and 13% in the SMILe–ICM group (hazard ratio 0.58, 95% CI: 0.26–1.28; *p* = 0.18). Thereafter, the curves continued to diverge, with a more pronounced separation during the subsequent months (the hazard ratio for GvHD after day 100 was 0.47, 95% CI: 0.25–0.88; *p* = 0.02). See Fig. 5a and Supplementary Fig. 2). Fifty-one patients showed no grade of acute GVHD (UCG:18; IG:33). Among those who developed acute GvHD, 28 reached a maximum severity of grade Ⅰ (UCG:13; IG:15), 34 grade II (UCG: 22/IG:12), 29 grade III (UCG:17/IG:12), and ten grade IV (UCG:6/IG:4). The mean time to onset was 7.8 months in the UCG and 8.2 in the SMILe–ICM group. Five patients were censored due to dropping out of the study; two more had competing events, receiving donor lymphocyte infusions 2–3 weeks before developing acute GvHD, and another two were censored because they had received donor lymphocyte infusions but could not say exactly when. In a sensitivity analysis, including the latter two as GvHD cases did not meaningfully alter the hazard ratio. A sensitivity analysis by center showed that both estimates had the same directionality, although somewhat more pronounced at the USB center HR = 0.31 was (95% CI: 0.14–0.67; *p* = 0.003), compared to the UKF center (HR = 0.87, 95% CI:0.46–1.66; *p* = 0.67).

The study-wide first-year cumulative incidence of chronic GvHD was 20.4% (33 patients, Fig. 5b). The incidence did not differ significantly between the study groups (*p* = 0.64). Ninety-seven patients did not develop any grade of chronic GvHD (UCG:43/IG:54). Of those who did, 22 reached a maximum severity of grade Ⅰ (UCG:16/IG:6), fourteen grade II (UCG:9/IG:5) and nineteen grade III (UCG:8/IG:11).

## Discussion

This study extends the initial SMILe–ICM effectiveness analyses across two alloSCT centers. As the first comprehensive evaluation of this care model’s secondary outcomes, it provides encouraging signs, particularly regarding *survival* and *timing and frequency of re-hospitalizations* [22, 23]. We now extend the evidence base by examining *patient-reported treatment and self-management burden*, *HRQOL*, and *clinically assessed GvHD*.

Compared to the UCG, the lower first-year cumulative incidence of grades II–IV acute GvHD in the SMILe–ICM group is one of this study’s most clinically relevant findings. In the SMILe–ICM group, the cumulative incidence of grades II–IV acute GvHD was 34.2%, compared with 55.3% in the UCG (HR 0.54, 95% CI 0.33–0.88, *p* = 0.01), indicating a lower incidence over the first post-transplant year. Contemporary studies typically report 28–40% cumulative incidences of these acute GvHD grades [33]. However, these estimates often reflect only the first 100 days post-alloSCT. By covering the entire first post-transplant year, we also included late-onset acute GvHD, including events occurring during the clinically important period of immunosuppressive tapering [34]. As our cumulative incidence estimates are expected to be higher owing to our longer observation period, direct comparisons should be interpreted with caution. Still, even with this longer observation period, the cumulative acute GvHD incidence observed in the SMILe–ICM group fell within the range reported in contemporary cohorts for much shorter observation periods. The cumulative incidence observed in the UCG is consistent with reports that also included late-onset acute GvHD [34].

Although ePRO data were reviewed according to predefined thresholds and a monitoring and response protocol, individual threshold events were not systematically linked to subsequent APN or physician assessments, treatment modifications, or time to treatment initiation. Therefore, it cannot be determined whether earlier recognition or treatment of emerging GvHD contributed to the lower cumulative incidence observed in the SMILe–ICM group. Earlier recognition, timely multidisciplinary assessment, and subsequent treatment adaptation should therefore be considered plausible but untested pathways rather than demonstrated mechanisms.

In contrast, chronic GvHD incidence did not differ significantly between groups over time. Admittedly, interpretation remains limited by one-year follow-up period: at study completion, the cumulative incidence curve had not yet plateaued. Compared to acute GvHD, chronic GvHD commonly develops later and is influenced predominantly by complex immunological and transplant-related factors, which are less likely to respond to supportive survivorship interventions alone [35]. Nevertheless, as this study was not powered for GvHD-related endpoints and we cannot exclude residual confounding by transplant-related risk factors, the GvHD findings should be interpreted cautiously, while explanations regarding the underlying mechanisms remain hypothesis-generating.

The *treatment burden* findings are consistent with a potential systems-of-care pathway, with the SMILe–ICM influencing aspects of survivorship care directly targeted by the intervention. In oncology, treatment burden is increasingly recognized as a high-value patient-reported outcome, reflecting not only the physical demands of treatment but also the cognitive, organizational, and emotional workload associated with managing complex care [36]. The most consistent intervention effects were observed in domains related to symptom management, self-management, and healthcare delivery, namely physical and mental fatigue, monitoring health, exercise or physical therapy, and difficulties with healthcare services. These domains directly reflect the SMILe–ICM’s core components of continuity of care, care coordination, and structured self-management support. Previous studies have demonstrated that structured nursing support can reduce the perceived workload of self-monitoring by helping patients prioritize relevant symptoms, interpret bodily signals, and avoid unnecessary vigilance [37]. Similarly, fatigue is among the most prevalent and burdensome symptoms experienced by cancer survivors and requires regular assessment, patient education, behavioral support, and exercise interventions, all of which represent core components of APN-led survivorship care [38].

Fragmented care pathways, poor inter-provider communication, and unclear responsibilities substantially increase patients’ workload [39]. Related literature indicates that care coordination and navigation support—core elements of integrative care models—can substantially reduce this burden by providing a single point of contact, facilitating communication, and addressing problems before they escalate [40]. The sustained effects we observed in this domain are consistent with previous findings in supportive cancer care and follow the SMILe–ICM’s proposed systems-of-care pathway.

Contrastingly, no sustained effects were observed in domains determined predominantly by structural or healthcare system factors, e.g., financial burden. This pattern further supports our interpretation that the intervention primarily influenced aspects of survivorship care amenable to coordinated care processes and behavioral support, i.e., factors within the scope of an APN-led integrated care model [41].

This study also evaluated the impact of the SMILe–ICM on HRQoL using the EQ-5D-5 L over a 12-month follow-up period. Contrary to our hypothesis, no statistically significant differences between the SMILe–ICM and usual care groups were observed across the EQ-5D dimensions, index values, or VAS scores. Importantly, baseline EQ-5D-5 L index values (0.84) were already close to normative values reported for the German general population (0.85–0.90) [30], suggesting relatively well-preserved HRQoL, leaving limited room for measurable improvement on a generic preference-based instrument. Thus, the absence of statistically significant between-group differences may reflect a ceiling effect rather than a lack of SMILe–ICM intervention benefit.

These findings support the proposed systems-of-care pathway, as the intervention was designed to improve continuity of care, care coordination and self-management rather than generic health status. Consequently, improvements may first become apparent in outcomes related more closely to the care experience, e.g., treatment burden, while changes in generic HRQoL may remain difficult to detect using a preference-based instrument with relatively high BL values.

Both groups experienced the expected decline in HRQoL during early recovery after transplantation, followed by improvement over the remainder of the first year. Although differences between groups did not reach statistical significance, consistently higher EQ-5D index values in the intervention group suggest a trend towards faster recovery that may not have been detectable given the limited sample size and the sensitivity of the EQ-5D-5 L.

A similar pattern was observed for the EQ-5D VAS, with both groups reporting marked improvements over time and consistently higher self-rated health in the intervention group. The higher VAS scores support the overall direction of the treatment burden findings. Together, these results suggest that the SMILe–ICM may primarily improve patients’ experience of living with and managing the demands of survivorship care, rather than substantially altering generic health status. This interpretation is consistent with previous observations that supportive care interventions often have a greater impact on patients’ perceived well-being, coping and adaptation than on preference-weighted measures of functional health [42].

### Strengths and limitations

Strengths of this study include the first comprehensive evaluation of patient-reported and clinical secondary outcomes of an integrated APN-led survivorship care model across two linked randomized controlled trials. Limitations include the exploratory nature of this secondary analysis, which was not powered for these outcomes, and that the proposed systems-of-care pathway remains hypothesis-generating because it was not directly evaluated. The randomized study design supports comparability between the groups and reduces the risk of systematic baseline differences. However, relevant transplant-related GvHD risk factors, including donor type, HLA matching, stem-cell source, and GvHD prophylaxis, were not available in the harmonized dataset. Chance imbalances in these factors therefore cannot be excluded, particularly given the sample size. Moreover, the trial was not designed to investigate the mechanisms underlying GvHD occurrence, and the observed association should therefore be interpreted cautiously. Future studies should prospectively evaluate threshold events identified through ePRO monitoring, subsequent APN and physician assessments, treatment decisions, and time to treatment initiation to determine whether and how integrated survivorship care may influence GvHD outcomes.

Finally, differences in usual-care pathways between the two centers, including the possibility of post-alloSCT discharge to a rehabilitation clinic at UKF, may have influenced treatment burden, monitoring, or other outcomes and should be considered when interpreting the pooled finding.

## Conclusions

This study extends the evidence generated by the international SMILe research program by showing that the previously observed signs of improved *survival*, reduced *re-hospitalization*, and *cost-effectiveness* accompanied reductions in both *treatment burden* and *incidence of clinically relevant acute GvHD*. These findings are unlikely to reflect a direct biological intervention effect, but do support a systems-of-care mechanism whereby integrated eHealth-facilitated APN-led survivorship care improves continuity of care, timely recognition of complications, care coordination, and self-management. Confirmation via adequately-powered multicenter studies is warranted to establish this model’s effectiveness and to further elucidate the mechanisms supporting improved long-term post-alloSCT outcomes.

## Supplementary Information

Below is the link to the electronic supplementary material.

Supplementary Material 1

## Data Availability

The datasets generated and analysed during the current study are not publicly available because they contain sensitive clinical data from a small and highly vulnerable patient population, which may compromise participant confidentiality despite de-identification. Data may be made available from the corresponding author on reasonable request, subject to institutional approval, applicable data protection regulations, and ethics committee requirements.
