# Supplementary material for: Evaluating the effectiveness of the eHealth-faciLitated SMILe–integrated care model on treatment burden, quality of life and graft-versus-host disease after allogeneic stem cell transplantation: results of two European randomized controlled implementation studies
Source: Ann Hematol. 2026 Oct 24;105(11):484. doi: 10.1007/s00277-026-07333-1 (PMC13696606; doi:10.1007/s00277-026-07333-1)
Supplement: Supplementary file 1 [file 277_2026_7333_MOESM1_ESM.docx]

**Supplementary Tab 1.** Predefined monitoring thresholds and APN response protocol for the remote monitoring within the SMILe–ICM

| **Parameter** | **Scoring** | **Watchful waiting** | **Contact patient** | **Predefined APN responses** |
| --- | --- | --- | --- | --- |
| **Wellbeing** | 1-10 | ≤3 | >3 | Call or wait until next visit if scheduled within next three days for clinical assessment |
| **Weight** | kg | 5% variation | ≥10% variation | Call or wait until next visit if scheduled within next three days for clinical assessment |
| **Systolic blood pressure** | mmHg | 70-140 | ≥140  < 70 | Call and take clinical assessment, ask patient to measure blood pressure/heart frequency again after 30 min. Refer to local GP/oncologist or discuss with transplant physician if it remains below/above thresholds |
| Diastolic blood pressure | mmHg | 50-100 | ≥100 mmHg  ≤50 mmHg |  |
| **Heart frequency** | **beats/min** | NA | ≥130 bpm or <50 bpm |  |
| **Temperature** | °C | 37.5–38.0 °C | >38.0 °C | Call and take clinical assessment. Discuss with transplant physician. |
| **Immunosuppressive medication taken** | yes, no,  I didn’t take dose | NA | Missed dose | Call and take clinical assessment. Ask for barriers, offer support. |
| **Other medication taken as prescribed** | yes, no, I didn’t take medication | NA | Missed doses |  |
| **Skin rash** | Number of hands = % of body surface (1 hand = 1%) | NA | > 1 hand | Call and take clinical assessment. Ask for related symptoms. Discuss with transplant physician. |
| **Signs of bleeding** | 1-10 | <3 | ≥3 |  |
| **Mouth/throat sores** | 1-10 | <3 | ≥3 |  |
| **Reduced appetite** | 1-10 | ≤7 | >7 |  |
| **Nausea** | 1-10 | <3 | ≥3 |  |
| **Vomiting** | Number of times per day | <3 | ≥3 |  |
| **Diarrhea** | Number of times per day | <3 | ≥3 |  |
| **Shortness of breath** | 1-10 | <3 | ≥3 |  |
| **Coughing** | 1-10 | <7 | ≥7 |  |
| **Pain urinating** | 1-10 | <3 | ≥3 |  |
| **Pain** | 1-10 | <3 | ≥3 |  |
| **Fatigue** | 1-10 | ≤7 | >7 | Discuss in next planned visit and offer support |

**Supplementary Tab 2**: EG-5D-5L Mean Index Values weighted according to the German Value Set

|  | **EQ5D Utility** | | **EQ-VAS** | |
| --- | --- | --- | --- | --- |
| **Time point** | **UCG**  **N; mean (SD)** | **SMILe–ICM**  **N; mean (SD)** | **UCG**  **N; mean (SD)** | **SMILe–ICM**  **N; mean (SD)** |
| BL (Baseline) | 74; 0.83 (0.13) | 74; 0.85 (0.13) | 74; 56.45 (23.26) | 74; 62.45 (22.84) |
| T1 (+30 days) | 62; 0.81 (0.13) | 71; 0.82 (0.13) | 66; 59.95 (17.39) | 72; 62.51 (19.73) |
| T2 (+60 days) | 64; 0.83 (0.13) | 70; 0.84 (0.14) | 65; 61.83 (16.14) | 70; 67.37 (20.21) |
| T3 (+90 days) | 63; 0.82 (0.16) | 66; 0.83 (0.18) | 63; 64.13 (17.78) | 65; 68.03 (21.94) |
| T4 (+120 days) | 61; 0.83 (0.12) | 67; 0.87 (0.10) | 61; 65.46 (17.25) | 65; 74.20 (16.08) |
| T5 (+150 days) | 56; 0.84 (0.10) | 66; 0.88 (0.10) | 58; 66.78 (18.86) | 66; 73.38 (20.57) |
| T6 (+180 days) | 56; 0.87 (0.11) | 66; 0.87 (0.11) | 57; 70.89 (18.72) | 66; 73.67 (19.76) |
| T7 (+240 days) | 55; 0.86 (0.12) | 61; 0.89 (0.08) | 55; 69.91 (20.09) | 61; 78.98 (14.07) |
| T8 (+300 days) | 53; 0.85 (0.11) | 61; 0.88 (0.10) | 52; 73.19 (18.18) | 61; 78.41 (16.35) |
| T9 (+365 days) | 50; 0.87 (0.10) | 58; 0.88 (0.10) | 51; 77.06 (17.36) | 58; 79.60 (15.92) |

**Supplementary Fig.1**: EQ-5D-5L – Visual Analogue Scale over all time-points per group

**Supplementary Fig. 2:** Cumulative incidence of new-onset acute GvHD occurrences post d+ 100

 *Note:* Cumulative incidence of acute and chronic GvHD over time in the UCG (blue line) and SMILe-ICM/IG (red line). Shaded areas indicate 95% confidence intervals. The vertical line indicates the landmark of day 100.
